# Systematical Characterization of the *AT-Hook* Gene Family in *Juglans regia* L. and the Functional Analysis of the *JrAHL2* in Flower Induction and Hypocotyl Elongation

**DOI:** 10.3390/ijms24087244

**Published:** 2023-04-14

**Authors:** Peng Jia, Jiale Liu, Rui Yan, Kaiyu Yang, Qinglong Dong, Haoan Luan, Xuemei Zhang, Han Li, Suping Guo, Guohui Qi

**Affiliations:** College of Forestry, Hebei Agricultural University, Baoding 071000, Chinayangkaiyu2023@163.com (K.Y.);

**Keywords:** AT-hook gene family, *Juglans regia*, hypocotyl, flowering, Phylogenetics

## Abstract

AT-hook motif nuclear localization (AHL) proteins play essential roles in various plant biological processes. Yet, a comprehensive understanding of *AHL* transcription factors in walnut (*Juglans regia* L.) is missing. In this study, 37 *AHL* gene family members were first identified in the walnut genome. Based on the evolutionary analysis, *JrAHL* genes were grouped into two clades, and their expansion may occur due to segmental duplication. The stress-responsive nature and driving of developmental activities of *JrAHL* genes were revealed by *cis*-acting elements and transcriptomic data, respectively. Tissue-specific expression analysis showed that *JrAHLs* had a profound transcription in flower and shoot tip, *JrAHL2* in particular. Subcellular localization showed that JrAHL2 is anchored to the nucleus. Overexpression of *JrAHL2* in Arabidopsis adversely affected hypocotyl elongation and delayed flowering. Our study, for the first time, presented a detailed analysis of *JrAHL* genes in walnut and provided theoretical knowledge for future genetic breeding programs.

## 1. Introduction

The *AT-hook motif nuclear localized* (*AHL*) gene family encodes the conserved transcription factor that widely distributed in plants species [1]. The AHL proteins are generally distinguished by two characteristic domains: the AT-hook motif and the Plant and Prokaryote Conserved (PPC) domain. The AT-hook motif was first described as a high mobility group (HMG) non-histone chromosomal protein HMG-I/Y [2]. The AT-hook motif contains conserved core sequence ‘Arg-Gly-Arg’ that can bind to the minor groove of the AT-rich B form of DNA [3]. AT-hook motifs can be divided into two categories according to the conserved amino acid sequence: Type-I AT-hook motif contains ‘Gly-Ser-Lys-Asn-Lys’ consensus sequences at the end of the ‘Arg-Gly-Arg’ core, while the Type-II contains ‘Arg-Lys-Tyr.’ The PPC domain, also known as the Domain of Unknown Function #296 (DUF296), is also found in, archaea, prokaryotes, and higher plants. The PPC domain was involved in the physical interaction with other AHL (or themselves) or nuclear proteins [1,4]. According to sequence differences, the PPC can be divided into two categories: Type-A and Type-B. Type-B contains the conserved core ‘Phe-Thr-Pro-His’ in the upstream region, while the core sequence within the PPC domain in Type-A is more variable (‘Leu-Arg-Ser-His’ or ‘Leu-Arg-Ala-His’, etc.) [5]. The *AHL* genes in land plants have been grouped into Clades based on the phylogenetic relationship: Clades-A and Clades-B. The Clade-A *AHLs* are intron-free and encode proteins containing only one Type-I AT-hook motif and Type-A PPC domain. The Clade-B *AHLs* contain introns, and Clade-B AHL proteins can be further divided into two types: one type of AHLs contains a Type-II AT-hook motif and a Type-B PPC domain, and the other type of AHLs contains two AT-hooks (Type-I and Type-II) and a Type-B PPC domain [6].

The AHL gene family plays vital roles in plant growth, development, and stimulus response. In Arabidopsis (Arabidopsis thaliana), the biological functions of several AHLs have been extensively studied. A number of AHL genes redundantly inhibit the elongation of hypocotyl, such as AHL22 [7], SOB3/AHL29 [6,8], and ESC/AHL27 [9]. *AHL18*, which is involved in the regulation of the length of the proliferation domain and the division in the root apical meristem, and inhibiting primary root growth and lateral root development [10]. In *M. truncatula*, the *MtAHL1* and *MtAHL2* were involved in the formation of nitrogen-fixing nodules [11].

The *AHL* gene is related to regulating flowering time and reproductive development differently. The TEK/AHL16 directly binds to the floral suppressor gene FLC’s repressive regulatory region, leading to its transcriptional silencing. Knockout of the *TEK/AHL16* activates a variety of transposons, and up-regulate the expression of floral suppressor gene *FLC* through epigenetic regulation (histone acetylation and DNA methylation), thereby delaying flowering [12]. TEK/AHL16 also contributes to nexine formation and pollen wall development by directly binding to the nuclear matrix attachment region (MAR) and the promoter of *AGP6*, an arabinogalactan protein coding gene [13,14]. In addition, overexpressing *AHL* delays flowering by inhibiting *FT* expression [7,15]. AHL22 binds to the AT-rich sequence in the *FT* locus and then alters the structural design of the chromatin region by regulating both histone acetylation and methylation [15]. *AHL18*, which is in the same phylogenetic clade as *AHL22*, conferred a similar late-flowering phenotype when overexpression in Arabidopsis to that of *AHL22* [7]. AHLs were also reported to be involved in hormone-related pathways. SOB3/AHL29 inhibited PIF4 protein accumulation, thereby reducing the binding of PIF4 to target loci involved in hormone transport, homeostasis, or response such as auxin, brassinosteroid, and ethylene [16]. SOB3/AHL29 also targeted downstream genes associated with auxin-related genes, such as *YUCCA8* and *SAUR19* [8]. AGF1/AHL25 regulated gibberellins homeostasis in Arabidopsis by targeting the GA 3-oxidase gene AtGA3ox1 [17]. In addition, AHLs participated in the biotic and abiotic stress responses. AHL13, AHL15, AHL19, AHL20, and AHL27 were reported to implicate in PAMP-triggered immunity [18,19,20]. Overexpression of AHL20 and its closely related family members (AHL15, AHL19, and AHL27) in protoplasts inhibited the innate immune response [19]. Especially in Arabidopsis, AHL13 regulated key factors in the jasmonic acid (JA) pathway and affected immunity toward Botrytis cinerea and Pseudomonas syringae pathogens [20]. AHL10 played a critical in response to low-water potential stress, and in the regulation of auxin- and jasmonic acid -related gene expression [21]. AtAHL4 was involved in the regulation of lipid catabolism during seedling establishment [22] and of root xylem development [4]. Interactions between AHL proteins are also crucial for their functions. In Arabidopsis roots, the AHL3 physically interacted with AHL4 and facilitated intercellular trafficking [23]. In maize (*Zea mays*), the AT-hook protein BAF1 is capable of forming homodimers and heterodimers with other members of the AT-hook family, controlling the formation of maize ears [24].

In recent years, the AHL gene family has been identified and analyzed in several species, such as Arabidopsis, soybean (*Glycine max*) [25], cotton (*Gossypium raimondii*) [26], *Brassica napus* [27], grape (*Vitis vinifera*) [28], carrot (*Daucus carota subsp. sativus*) [29], and so on. Walnut (*Juglans regia* L.) is an economically vital nut trees in China and other temperate parts of the world, but the long period before flowering and bearing fruiting after seed germination limits the progress of walnut breeding work. In addition, functional characterization of AHL genes in walnut is still missing. In this study, the AHL gene family was characterized in walnut based on the high-quality genome data [30]. The chromosomal locations, phylogenetic relationships, gene expansions and structures, conserved domains, putative protein interaction, and cis-elements in the promoter region were analyzed. Furthermore, the *JrAHL2*, a Clade-A member highly expressed in flower, was isolated, its protein subcellular localization was analyzed, and its functions involved in floral transition and hypocotyl elongation were dissected. The current study will provide a theoretical basis for further investigation of the functions of the *JrAHL* gene family, and an improved understanding of the molecular mechanisms underlying its role in regulating growth and development in walnut.

## 2. Results

### 2.1. AHL Genes in Walnut

The HMMscan was executed against the protein databases using the PPC/DUF296 (PF03479) and AT-hook as queries. After manual checking, the 37 unique *AHL* genes were obtained from walnut (*Juglans regia* L.). The *JrAHL* genes were named according to their chromosomal locations (*JrAHL1*–*JrAHL37*) (Table 1). The gene lengths of *JrAHL* genes ranged from 667 bp to 7791 bp, which encode polypeptides from 221 to 406 amino acids with predicted molecular weights ranging from 20.04 kDa to 42.63 kDa. The theoretical pI ranged from 5.08 to 10.61. Approximately 92% of JrAHL proteins (except for JrAHL10/11/33) were predicted to be located in the nucleus (Supplementary Table S2), consistent with the previous report [1].

### 2.2. Phylogenetic Analysis of JrAHL Genes

**Figure 1:**
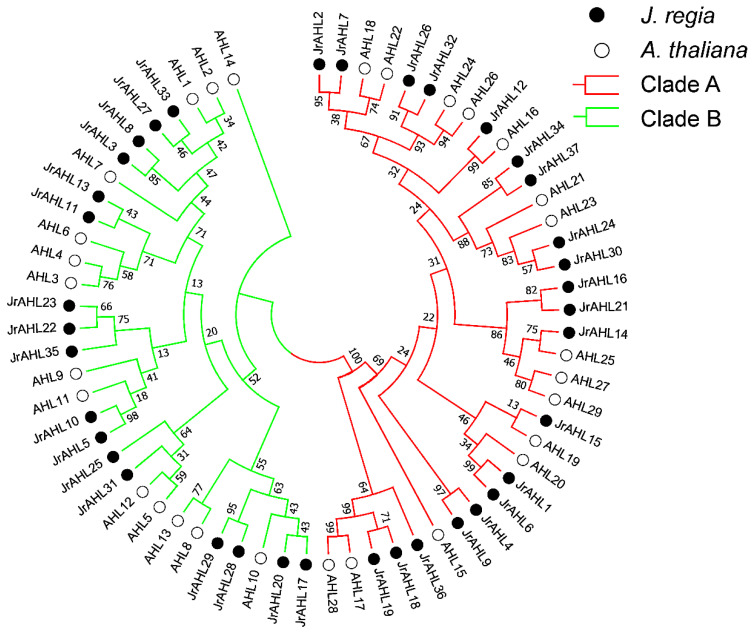
Neighbor-joining tree representing phylogenetic relationships among *AHL* genes from walnut and Arabidopsis. The evolutionary history was inferred using the Neighbor-Joining matrix-based method and are in the units of the number of amino acid substitutions per site. The rate variation among sites was modeled with a gamma distribution. Evolutionary analyses were conducted in MEGA7.

**Figure 2:**
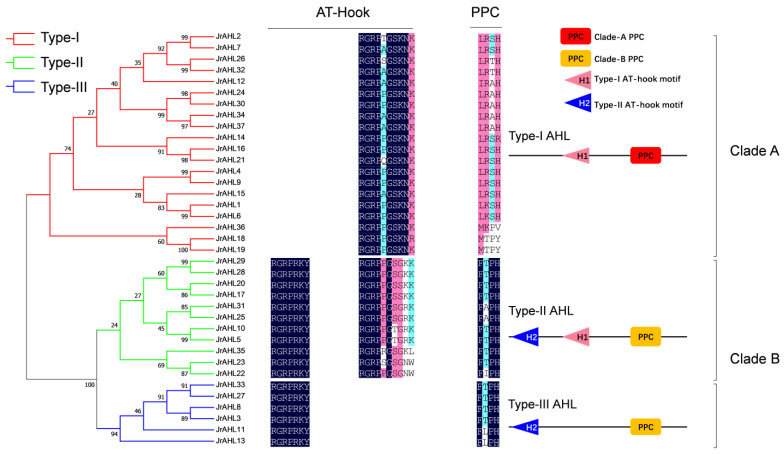
Phylogenetic (**left panel**) and sequence alignment (**right panel**) analysis of the walnut AHL proteins. The obtained phylogenetic tree by MEGA7 is shown on the left. Multiple sequence alignment analysis was performed using DNAMAN software, and the conserved domain is displayed on the right.

To infer the evolutionary relationship among the *JrAHL* proteins, phylogenetic analysis was performed on the full-length protein sequences. The result showed that the JrAHL proteins in walnut could be divided into two clades, as in Arabidopsis (Figure 1)-Clade-A and Clade-B. JrAHL were further classified into Type-I, Type-II, and Type-III (Figure 2 left panel), based on the presence and combination of the two characteristic functional units-the PPC domain and the AT-hook motif (Figure 2 right panel). Clade-A contained 20 JrAHLs, and it was also classified into Type-I, harboring the Clade-A PPC domain and the Type-I AT-hook motifs in their putative protein sequence (Figure 2 right panel). The higher abundance of Type I members (54%) in walnut is also consistent with observations in other land plants [25,27,31]. There were 17 JrAHL members in Clade-B, which all harbored the Clade-B PPC domain. Clade-B was further divided into two types: 11 Type-II members and 6 Type-III members. Type-II members contained Type-I and Type-II AT-hook motifs, whereas the Type-III JrAHLs only contained the Type-II AT-hook motif (Figure 2 right panel).

### 2.3. Gene Structure and Duplication of JrAHLs

**Figure 3:**
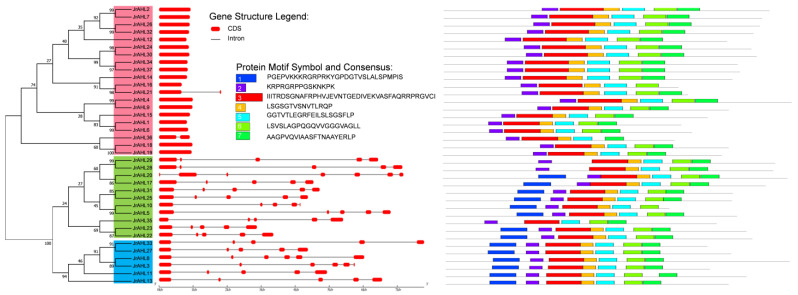
Gene structure analysis and conservative motif prediction of the AT-hook motif gene family in walnut. The gene structure map was obtained with the GSDS online tool (**left**). The *x*-axis shows the inferred length of the different genes (5′ to 3′) and their respective CDS (red). Domain structure and motif were elucidated with the MEME platform (**right**).

The distribution of introns and exons was investigated to explore the diversity of gene structure. The structures of *JrAHL* genes could be divided into two types, one contained no or only one intron, and the other contained multiple introns (Figure 3 left panel). All Type-II and Type-III *JrAHLs* contained four to five introns, while the *JrAHLs* in Type-I were intronless except for *JrAHL21* and *JrAHL26*. Furthermore, the number and arrangement of introns are relatively conserved within the same clade, implicating the evolutionary similarity between these members. A total of seven conserved motifs in which two motifs (motif 1 and motif 2) have highly conserved ‘R-G-R-P’ amino acid sequences (Figure 3 right panel), which are the typical characteristic sequences of the AT-hook motif family.

**Figure 4:**
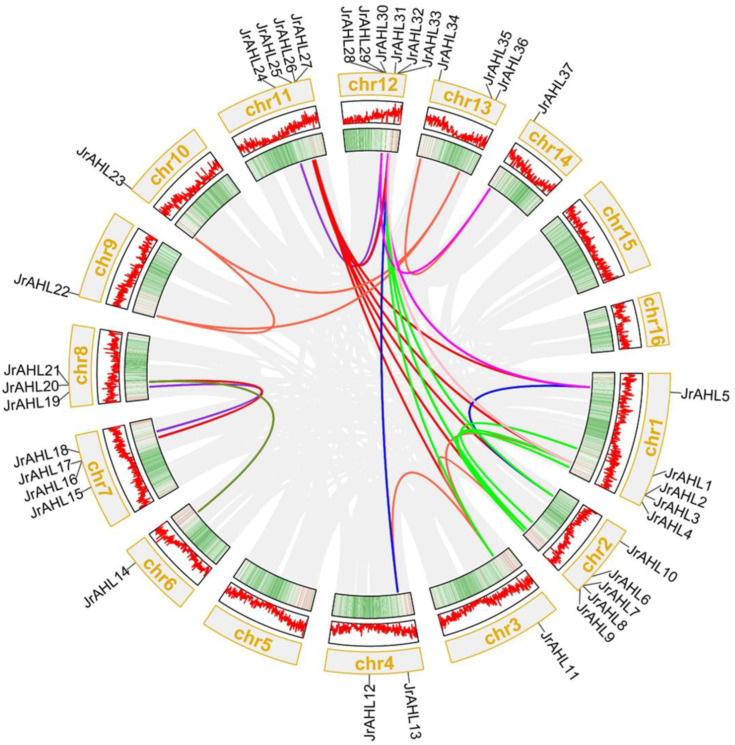
Chromosomal distribution and collinearity of walnut *AHL* genes. The gray lines in the background indicate the collinear blocks in the genomes of *J. regia*, while the colorful lines emphasize the segmental duplication of the walnut *AHLs*.

Gene duplication leads to the expansion of AHL family through segmental and tandem duplications [1]. We speculated the exact mechanism has driven the expansion of the *AHL* gene family in walnut. We analyzed gene duplication events in the *JrAHL* gene family. As shown in Figure 4, a total of 37 *JrAHL* genes were unevenly mapped onto 16 chromosomes of walnut. Chromosomes 00 and 14 did not contain duplicated genes, whereas Chromosome 12 had the highest number of duplications. More than twenty pairs of *JrAHL* genes, such as *JrAHL1/6*, *JrAHL4/9*, *JrAHL24/34*, and *JrAHL31/25*, were located in duplicated genomic regions. Duplicated pairs showed similar motifs (Figure 2), patterns of gene structure (Figure 3), and clustered tightly in the evolutionary tree (Figure 1), suggesting the *JrAHL* genes have duplicated segmentally during the evolution.

### 2.4. Cis-Acting Element Analysis of JrAHLs Promoter

**Figure 5:**
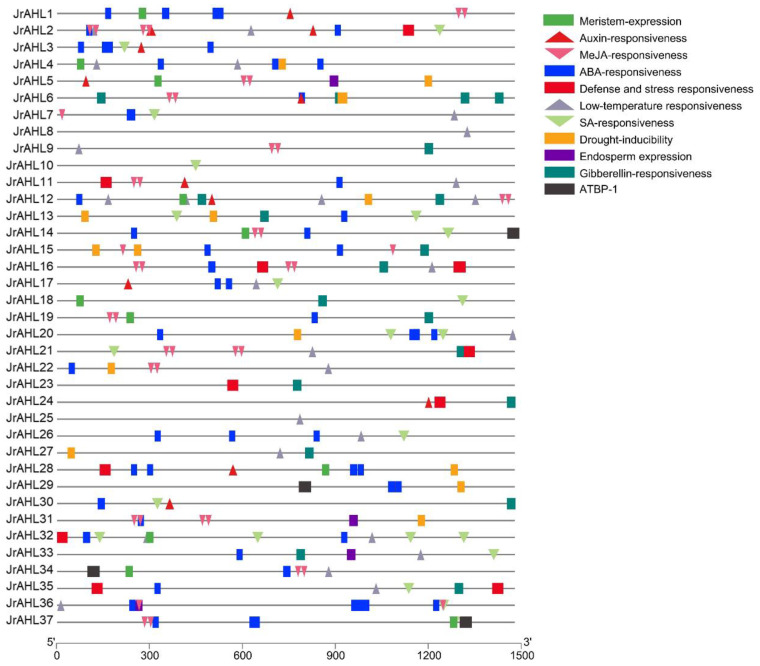
Predicted *cis*-elements in the *JrAHL* promoters. The 1.5-kb sequence upstream from the start codon of *JrAHL* genes was analyzed using the PlantCARE database. Different colored graphics represent different *cis*-acting elements.

The promoter is critical for transcriptional regulation because of its responsiveness to specific hormone levels and environmental cues and plays a vital role in the biological regulation of gene expression under stress. We investigated the possible regulatory mechanisms by searching the promoter region of 1500 bp upstream of the *JrAHL* gene family. Four categories of *cis*-elements were identified related to hormone response, stress response, tissue-specific expression, and transcription factor binding (Figure 5). Hormone response elements, such as gibberellin response element (GARE-motif), salicylic acid response element (TCA-element), abscisic acid-responsive element (ABRE), auxin-responsive (AUXRR-core), and MeJA-responsiveness (CGTCA-motif) were abundant in *JrAHL* promoter regions. Stress response elements, including defense responsive element (TC-rich repeat), low-temperature response (LTR), and drought response element (MBS), were discovered in most of the promoter regions of *JrAHL* genes. As for *cis*-acting regulatory elements related to tissue-specific expression, endosperm- and meristem-expression elements were found in 4 and 14 *JrAHL* promoter regions, respectively (Figure 5 and Figure S2). Four *JrAHL* genes contained the AT-rich element, which provided the binding site of ATBP-1, a class of AT-rich DNA binding proteins. These results imply that *JrAHL*s are involved in many biological processes.

### 2.5. Protein-Protein Interaction Analysis JrAHLs

**Figure 6:**
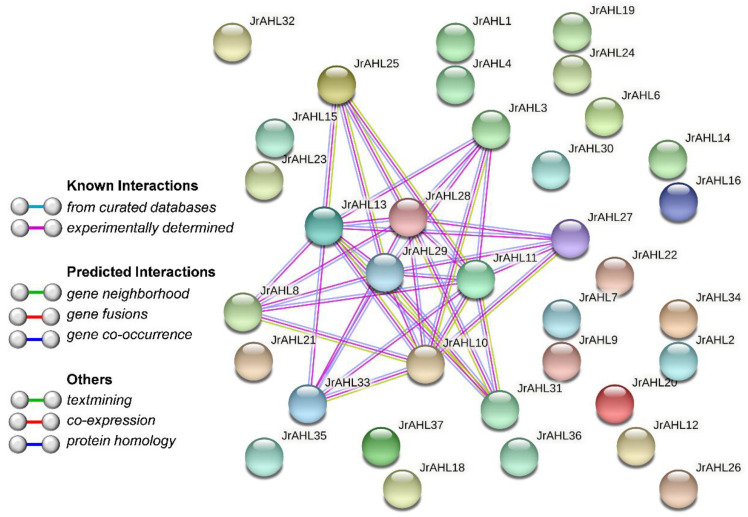
Predicted protein-protein interaction network of JrAHL protein. The network nodes represent proteins. The line color indicates the type of interaction evidence.

Members of the *AHL* family are partially involved in various plant biological processes via protein-protein interaction [23]. We constructed the protein interaction networks between the JrAHL proteins. Consistent with the previous report [16,24], the JrAHLs are shown to interact with each other (Figure 6). Additionally, JrAHL3/JrAHL8/JrAHL11/JrAHL13/JrAHL27/JrAHL33 were predicted in the networks, which were clustered with the physically-interacting proteins AHL3/AHL4 from Arabidopsis [23]. In *Poncirus trifoliata*, both PtrAHL14 and PtrAHL17 could interact with other proteins to target the same downstream genes [32]. However, the interaction between JrAHL proteins must be further verified in vitro and in vivo.

### 2.6. Gene Expression Patterns of JrAHLs

**Figure 7:**
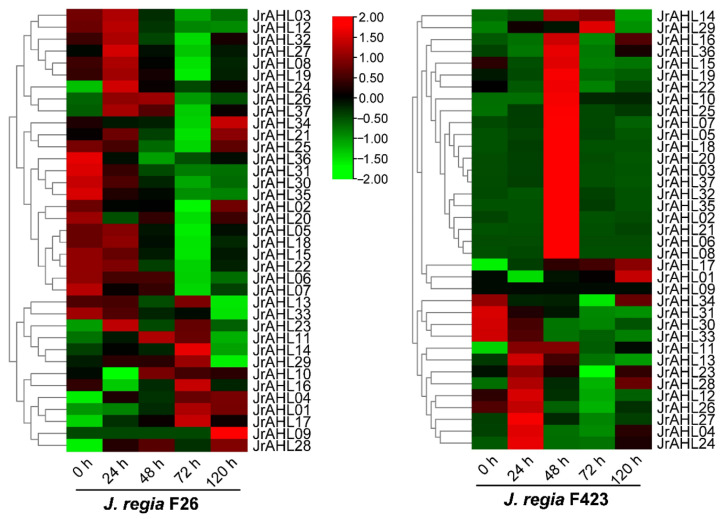
*JrAHL* gene expression profiles in F26 and the F423 fruits in response to anthracnose infection. The heat map was generated based on the transcriptomic data of the anthracnose-resistant F26 fruit bracts and anthracnose-susceptible F423 fruit bracts at five time points after infection with *C. gloeosporioides*.

**Figure 8:**
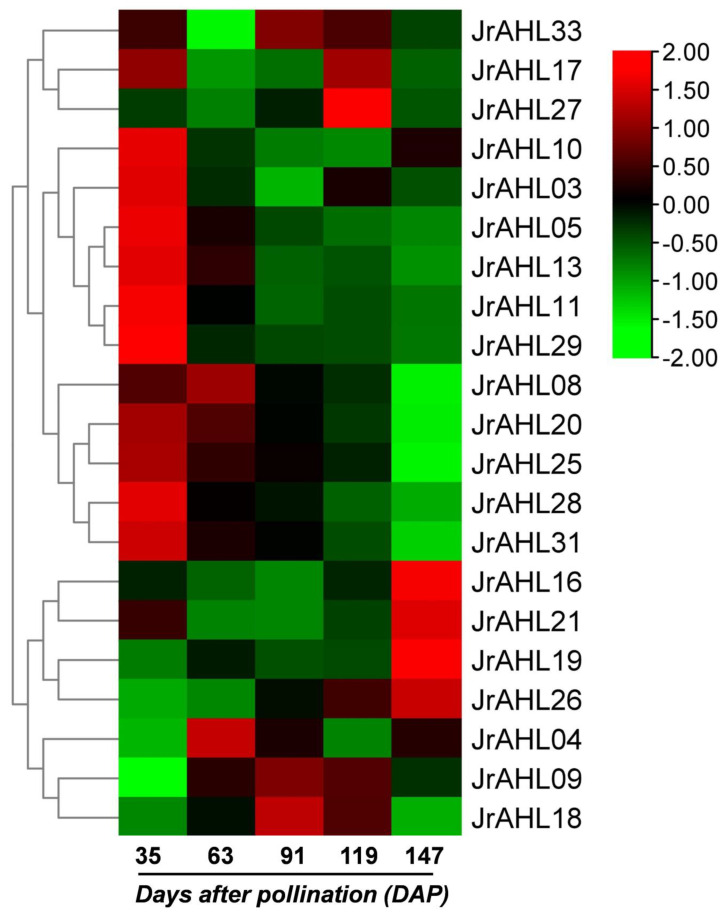
*JrAHL* gene expression profiles at different developmental stages of endopleura in walnut. The heat map was generated based on the transcriptomic data of the endopleura at different (35, 63, 91, 119, and 147) days after pollination (DAP).

**Figure 9:**
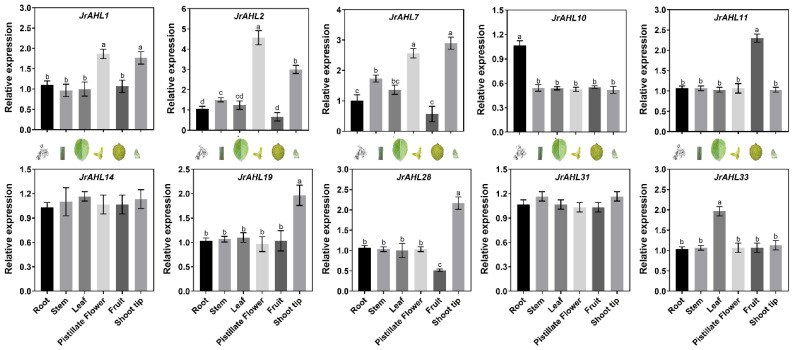
*JrAHL* gene expression profiles in different tissues. Each value represents the mean ± standard error of three replicates. Different letters (a, b, c, d) indicate significant differences, as determined by Student’s *t*-test at the 0.05 level.

The *JrAHLs* expression profiles of F26 (anthracnose-resistant) and F423 (anthracnose-susceptible) fruit bracts at five-time points after infection were analyzed based on the transcriptomic data. In the anthracnose-resistant F26, more than half of the *JrAHLs* gene members were up-regulated within 24 hours after inoculation. However, the up-regulation of these *JrAHL* genes was delayed in the susceptible F423 (Figure 7). This result suggested that *JrAHLs* are involved in the immune regulation of pathogens. Expression profiles of *JrAHLs* at different developmental stages of endopleura, one of the walnut’s most commercially essential organs, were analyzed. More than half of *JrAHL* genes showed high transcription levels in the early endopleura development stage (Figure 8). The tissue-specific expression profile of some *JrAHL* genes from different clades was determined by real-time quantitative PCR (Figure 9). *JrAHL1*, *JrAHL2*, *JrAHL7*, *JrAHL19*, and *JrAHL28* were highly expressed in the shoot tip. *JrAHL1*, *JrAHL2*, and *JrAHL7* also showed high expression levels in pistillate flowers. Two genes were preferentially expressed in the vegetative organs, such as *JrAHL10* in root and *JrAHL33* in leaf. The *JrAHL11* is highly expressed in fruit. In addition, *JrAHL14* and *JrAHL31* showed no tissue-specific expression.

### 2.7. Gene Cloning and Subcellular Localization of JrAHL2

**Figure 10:**
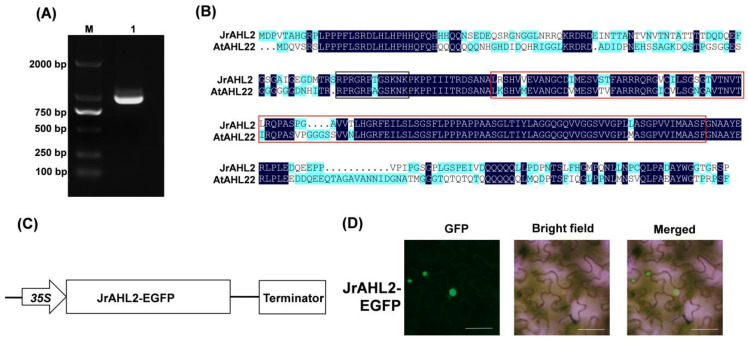
*JrAHL2* gene cloning and subcellular localization. (**A**) Gene cloning of *JrAHL2* coding region. (**B**) Putative protein sequence analysis of the cloned *JrAHL2* gene. (**C**) Schematic diagram of the *JrAHL2* overexpression vector for subcellular localization. (**D**) Subcellular localization of JrAHL2 in tobacco leaves.

*JrAHL2* was cloned from walnut and found to contain a 927-bp CDS (Figure 10A) that encodes a protein of 307 amino acids. Multiple sequence alignment analysis results showed that the JrAHL2 shared 59.75% sequence identity with AtAHL22. The amino acid sequence within the AT-hook motif and the large PPC/DUF296 domain are highly conserved (Figure 10B). JrAHL2 protein is predicted to be localized in the nucleus (Supplementary Table S2). JrAHL2 was fused to a green fluorescent protein (EGFP) (Figure 10C) and transiently expressed in tobacco leaf cells. The GFP signal was targeted to the nucleus (Figure 10D), suggesting that JrAHL2 functions as a transcription factor.

### 2.8. Functional Analysis of JrAHL2 in Transgenic Arabidopsis

**Figure 11:**
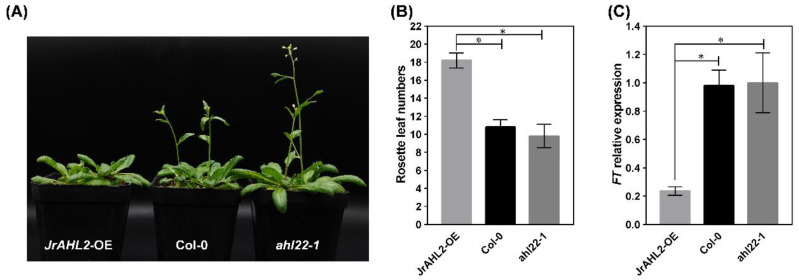
*JrAHL2* affects flowering in Arabidopsis. (**A**) Flowering phenotype of *JrAHL2*-overexpressing (*JrAHL2*-OE), wide type (Col-0), and *AtAHL* mutant (*ahl22-1*) Arabidopsis lines, respectively. (**B**) The number of rosette leaves at flowering. (**C**) The expression level of flowering-related gene *FT*. The columns represent the mean values of three replicates ± standard deviations. The asterisk (*) indicate significant differences, as determined by Student’s *t*-test at the 0.05 level.

**Figure 12:**
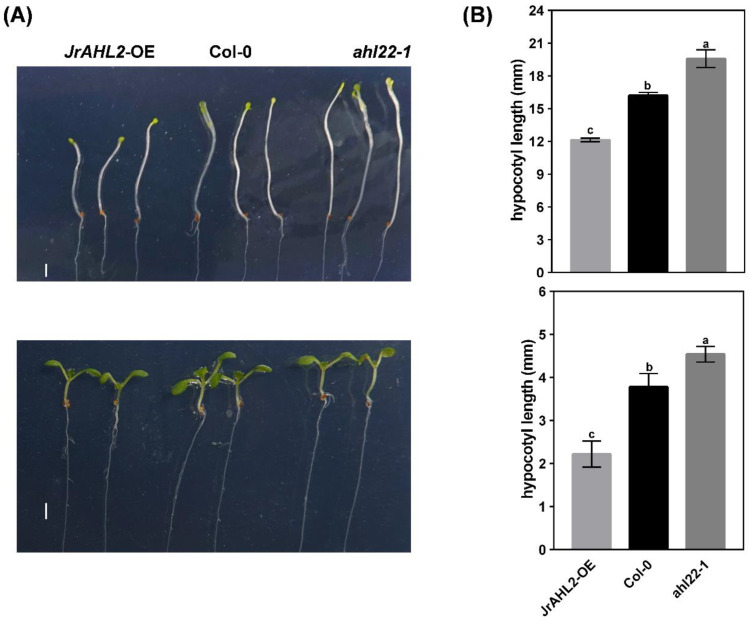
*JrAHL2* affects hypocotyl elongation. Samples were photographed (**A**), (**upper panel**) for darkness, and (**lower panel**) for normal photoperiod and the length of the hypocotyl was analyzed (**B**). Bar = 2 mm. The columns represent the mean values of three replicates ± standard deviations. Different letters (a, b, c) indicate significant differences, as determined by Student’s *t*-test at the 0.05 level.

To dissect the function of *JrAHL2*, we generated *JrAHL2*-overexpressing (OE) transgenic Arabidopsis plants. The homozygous *JrAHL2*-OE plants flowered significantly later than the WT controls (Figure 11A,B). Additionally, the flowering genes *AtFT* is expressed at a lower level in the *JrAHL2*-OE Arabidopsis plants (Figure 11C). Overexpression of *JrAHL2* also leads to a dominant-negative long hypocotyl phenotype under both long day and dark conditions (Figure 12). Thus, *JrAHL2* inhibits floral transition and hypocotyl elongation.

## 3. Discussion

### 3.1. Identification and Analysis of JrAHL Gene Family Members

The walnut (*Juglans regia* L.) is one of the most economically essential nut trees, and the research on walnut focused on flowering and molecular breeding in recent years. However, less attention was paid to walnut than other plants because of the problems in the walnut industry development, such as the long juvenile phase, difficulty in flowering, and hypocotyl elongation through the husk. The high-quality walnut genome has been published [30], providing a reference for systematic research on the genetic composition and potential function. To date, some gene families from walnut have been identified, such as the *Basic leucine zipper* (*bZIP*) [33] and *General Regulatory Factor* (*GRF*) [34] genes, which may be involved in the regulation of flowering. However, the role of the *AT-Hook Motif Containing Nuclear Localized* (*AHL*) gene family has not been explored in higher plants beyond herbaceous plants. In this work, 37 *JrAHL* members were identified in the walnut genome (Table 1). More member was identified in walnut compared to Arabidopsis (29 members) [6], probably because the walnut genome (540 Mb) is larger than that of Arabidopsis (125 Mb). The *JrAHL* members were divided into two Clades, based on the phylogenetic tree (Figure 1) and PPC/DUF domain (Figure 2) as reported in other species [1]. AHL proteins interact with each other through their PPC domain [35]. Thus, the variability increase in PPC domains may extend the range of biological functions. The Clade-B is further divided into two types based on the AT-hook motif, Type-II and Type-III. At the same time, the Clade-A is referred to as Type-I (Figure 2). Because the AT-hook motif is responsible for recognizing and binding a specific nucleotide sequence as well as changing the chromatin structure [36], it is, of course, possible that JrAHLs from the different branch can target different downstream genes. The sequences of conserved motifs (Figure 2), the gene structure (Figure 3), and the arrangement of distinct domains (Figure 3) and are highly consistent within each evolutionary branch. High consistency may be caused by segmental duplications (Figure 4) or the whole-genome duplication process [27], which are the major driving forces of *AHL* family evolution. Similarly, domain-swapping analyses showed that the N-terminus of AHL4 is required for its DNA-binding activity [4]. All *JrAHL* genes in Clade-B contain multiple exons, whereas all the Clade-A members are intronless (Figure 3). Introns are ubiquitous in eukaryotic genomes, which affect gene function by affecting gene expression regulation [37]. For example, the number of introns in soybeans certainly affects the expression of *AHL* genes [25]. In addition to regulating the rate of gene evolution [38], introns also provide specific functional elements for selective splicing and exon shuffling, and some functions of non-coding DNA [39], and in so doing allow the occurrence of diversity in JrAHLs protein accumulation, structures (Supplementary Figure S1), and physically interactions (Figure 6). Because the eukaryote genomes gained introns during or after prokaryote-eukaryote divergence [13], an early divergence occurred between the Type-I *JrAHLs* and the other two types (II and III) of *JrAHL* genes, and the *JrAHLs* of Clade-B in the walnut genome have diverged from Clade-A. Gene duplication, one of the most important features of plant genome structure, contributed to the evolution of novel functions [40] (Panchy et al., 2016). The ratio of non-synonymous to synonymous mutations in genes (Ka/Ks) of duplicated gene pairs was < 1, suggesting that purifying selection was the main source [40] of evolution for the *JrAHL* gene family. It is thus plausible that introns acquisition may cause the structural and functional changes of *JrAHLs* during duplication (Figure 3 and Figure 4).

In addition to introns, the promoter, another important non-coding region, plays a critical role in regulating gene transcription. The *cis*-acting elements of some duplicated gene pairs are not the same, such as *JrAHL1/6* and *JrAHL4/9*. These differences would allow the regulation of different aspects of plant development and the responses to hormones and stresses. Studies of promoters, especially *cis*-acting elements (*CREs*) are crucial for improving our fundamental understanding of gene regulation and function towards different environmental cues and stress. The *CREs* that regulate plant development and confer resistance stresses are found in the *JrAHL* promoters (Figure 5 and Figure S2). Stress-related hormone response elements, including ABA, MeJA, and SA, are widely distributed on multiple *JrAHL* promoters. Fifteen *JrAHLs* contained GARE-motif, especially *JrAHL6,* contains up to four of these elements, suggesting a cross-talk between *JrAHLs* and GA hormones, such as negative feedback regulation as previously reported [17]. Aux-responsive *CREs* are predicted in ten *JrAHL* promoters. Specifically, *JrAHL28* is homologous to *AHL10* in Arabidopsis, which regulates genes related to the auxin signaling pathway, suggesting a cross-talk between *JrAHLs* and auxin [8,21]. AHL also negatively regulates jasmonic acid (JA) levels and affects the gene expression in the JA pathway [20]. Notably, two AT-Hook proteins, PtrAHL14/17 in *Poncirus trifoliata,* were reported to modulate cold tolerance [32]. Adversity stress-related *CREs,* including low temperature, drought, and defense, suggest that *JrAHLs* are relevant to controlling these processes. Four *JrAHL* genes contained the AT-rich element, which provided the binding site of AT-rich DNA binding protein (ATBP-1) [41]. It is plausible that there is reciprocal transcriptional regulation among the *JrAHLs* family members, in addition to the predicted protein-protein interactions (Figure 6). In addition, tissue-specific elements such as endosperm- and meristem-expression (Figure 5 and Figure S2) suggested that *JrAHLs* regulate embryogenesis and meristem maturation like previously reported [35,42]. Specifically, *JrAHL17* contains both endosperm- and meristem-expression *CREs* and is homologous to *AtAHL10* from Arabidopsis, which affects the expression of the transcription factor *STM* (*Shootmeristemless*) required for meristem maintenance [21]. qRT-PCR revealed that the *JrAHL* genes containing meristem-expression tissue-specific elements in the promoter were highly expressed in the shoot tip (Figure 5 and Figure 9). Transcriptome data facilitate a comprehensive understanding of expression levels of multiple genes. Multiple *JrAHL* genes showed differential expression by analyzing the transcriptome data of two walnut materials after inoculation with *Colletotrichum gloeosporioides*. In response to *C. gloeosporioides* infection, more than half of the *JrAHL* genes were rapidly activated in F26 at the initial stage of inoculation. At the same time, the activation of *JrAHL* in F234 was relatively delayed, suggesting that *JrAHL* is involved in the immune regulation of pathogens and that early expression may confer high resistance to anthracnose in F26 fruits (Figure 7). In fact, there is already established evidence that *AHL* is involved in plant immunity and positively regulates plant resistance to pathogen infection [20,43]. More than half of the *JrAHL* genes are highly expressed at the beginning of endocarp development (Figure 8), including *JrAHL11*, which showed a profound expression in walnut fruit (Figure 9). *JrAHL11* was clustered into the same clade with the Arabidopsis *AHL4*, and the latter is involved in the regulation of lipid mobilization and fatty acid β-oxidation [22], suggesting that the *JrAHL11* plays an important role in fruit development. The three detected Type I *JrAHL* genes, *JrAHL1*, *JrAHL2*, and *JrAHL7*, were highly expressed in pistillate flowers and shoot tip (Figure 9). *JrAHL1* was homologous to the *AHL20*, *JrAHL2* and *JrAHL7* were homologous to the *AHL22* (Figure 1) in Arabidopsis, respectively. Both *AHL20* and *AHL22* were involved in controlling flowering time in Arabidopsis [7,15,44], suggesting that these three genes are involved in floral regulation.

### 3.2. JrAHL2 Delayed Flowering and Inhibited Hypocotyl Elongation

Hypocotyl elongation is an essential event in the photomorphogenesis of walnut seeds, and flowering is an important trait for economic forest species. The functions and mechanisms of *AHL* genes from Arabidopsis have been extensively studied, whereas the function dissection information about the specific *JrAHL* gene in walnut is still unavailable. The *JrAHL2* were highly expressed in the pistillate flower and was homologous to the Arabidopsis *AHL22*, which stimulates us to investigate the function of *JrAHL2* in controlling flowering and hypocotyl elongation. The transgenic Arabidopsis with constitutive expression of the *JrAHL2* gene was prepared. Overexpression of *JrAHL2* caused a significant inhibition of hypocotyl elongation, whereas the Arabidopsis *ahl22* (homologous to *JrAHL2*) mutant is longer in the hypocotyl length than wide type (Figure 10) under both long-day conditions and darkness. It reported that AHL22, SOB3/AHL29, ESC/AHL27 [6,7,8] are negative modulators of hypocotyl growth in Arabidopsis [9]. This result indicates that *JrAHL2* plays a negative role in controlling hypocotyl elongation and functions in both photomorphogensis and skotomorphogenesis. In addition to repressing the hypocotyl growth, *JrAHL2* also inhibits the transcription of *FT* and delays flowering (Figure 9). Nevertheless, no perturbations to flower phenotype and *FT* expression were recorded in the *ahl22* mutant (Figure 9), possibly because of extensive functional redundancy between *AtAHL22* and other *AHL* genes [15]. These results suggested that the *JrAHL2* has a much stronger effect on hypocotyl elongation than its effect on flowering regulation (Xiao et al., 2009). Additionally, despite the report that *AHL* regulates the expression of target genes, including *FT*, by directly binding to a specific nuclear MAR containing A/T-rich sequences within their promoter [15,32,44], the ubiquity of A/T-rich sequences in the promoter region makes it difficult to discriminate the elements specifically bound by *JrAHL2*. Thus, a systems approach such as ChIP-seq/qPCR could be used further to dissect the network of *JrAHL*.

## 4. Materials and Methods

### 4.1. Identification of the AHL Gene Family

For the discovery of the AHL family members in walnut, the high-quality common walnut (*Juglans regia* L.) reference genome data was downloaded [30]. The Hidden Markov Model scan (HMMscan) was used on the complete walnut protein database using PPC/DUF296 (PF03479) as well as AT-hook (PF02178) Pfam IDs obtained from the Pfam database (https://pfam.xfam.org/ (accessed on 7 January 2022)) [45] as queries. All non-redundant putative protein sequences were manually checked with the NCBI Conserved Domains Database (https://www.ncbi.nlm.nih.gov/Structure/cdd/wrpsb.cgi (accessed on 12 January 2022)). The unique sequences containing the AT-hook motif and the PPC domain were selected as putative JrAHLs. The online ExPASy program (http://www.expasy.org/tools/ (accessed on 12 February 2022)) was used to predict protein physicochemical characteristics. The online system Cell-PLoc (http://www.csbio.sjtu.edu.cn/bioinf/Cell-PLoc-2/ (accessed on 15 February 2022)) [46] was used to predict the subcellular localization of these JrAHL proteins.

### 4.2. Phylogenetic Analysis of AHL Proteins

A Neighbor-Joining tree was constructed to infer the phylogenetic relationship between the AHL proteins from Arabidopsis and walnut. Sequence alignment was carried out using ClustalW in MEGA7 [47] with default parameters. The 1000 Bootstrap replicates were applied to present the evolutionary history. Multiple alignments were also performed with DNAMAN software (LynnonBiosoft, San Ramon, CA, USA. version 8.0).

### 4.3. Genes and Protein Structure Analysis

The GSDS 2.0 (Gene Structure Display Server, http://gsds.cbi.pku.edu.cn) was utilized to draw the structure of JrAHL genes. MEME (http://meme-suite.org/ (accessed on 15 April 2022)) was employed for functional motif analysis. The three-dimensional structure of JrAHL proteins was predicted by the PHYRE server v2.0 (http://www.sbg.bio.ic.ac.uk/phyre2 (accessed on 22 April 2022)).

### 4.4. Collinearity Analysis

The OrthoMCL algorithm [48] was used to identify paralogous genes within the walnut genome, and then the MCScan [49] algorithm was applied to detect syntenic blocks containing walnut *AHL*. The Circos [50] was used to visualize the syntenic relationships between the walnut genomes. The Ka/Ks ratio was calculated to estimate the selection pressure among duplicated *JrAHL* genes as previously described [51]. All plugins are used in TB tool software (TBtools-II v1.108) [52].

### 4.5. Promoter Sequence Analysis

The 1.5-kb upstream region of the start codon of each JrAHL gene was retrieved. The online PlantCARE database (https://bioinformatics.psb.ugent.be/webtools/plantcare/html/ (accessed on 16 July 2022)) was used to identify the potential cis-acting elements.

### 4.6. Interactive Protein Network Analysis

The STRING database predicted the interaction network of AHL proteins (https://string-db.org/ (accessed on 25 July 2022)). All JrAHL proteins were used as the query to search for the walnut data in the STRING database. Both the functional and physical protein associations were included in the network. The minimum required interaction score was selected as medium confidence (0.400).

### 4.7. Expression Profiles of JrAHL Genes

Gene expression data from the anthracnose-resistant F26 and the anthracnose-susceptible F423 fruits in response to anthracnose (*Colletotrichum gloeosporioides*) infection were downloaded from the GEO database (https://www.ncbi.nlm.nih.gov/geo/ (accessed on 20 July 2022)) at accession GSE147083 [53]. Transcriptomic data of the walnut endopleura during its development was also downloaded from GEO database at accession GSE185230 [54]. The log2 transformed FPKM (Fragments Per Kilobase of exon model per Million mapped fragments) values were used for preparing the heatmaps. Root, stem, leaf, flowers, fruits, and shoot tip were taken, frozen immediately in liquid nitrogen, and stored at −80 °C until use.

### 4.8. Quantitative RT-PCR (qRT-PCR)

Total RNA was extracted from different tissues using the RNA extraction kit (OMEGA, Doraville, GA, USA). The RNA concentration was determined by spectrophotometer, and RNA integrity was verified by electrophoresis. First-strand cDNA was synthesized using the EasyScript^®^ One-Step cDNA Synthesis SuperMix (TransGen Biotech, China) following the manufacturer’s instructions. Primer pairs for qRT-PCR were designed using Primer Premier 6.0 (Premier Biosoft, San Francisco, CA, USA) (Supplementary Table S1). qRT-PCR was performed on a Bio-Rad CFX96 thermal cycler (Bio-Rad Laboratories, Hercules, CA, USA). The *18S* rRNA gene was used as a standard internal control. The relative expression levels were calculated by the comparative 2^−ΔΔCt^ method [55].

### 4.9. Gene Cloning, Vector Construction, and Subcellular Localization

The coding sequence (CDS) of the *JrAHL2* was cloned from the walnut pistillate flower’s cDNA and then inserted into the modified pCAMBIA2300-EGFP vector. Subcellular localization was carried out according to our previously published article [56]. The leaves of 5-week-old tobacco plants were infiltrated with the *Agrobacterium* strain GV3103 cells containing the recombinant vector. After 3 d, the green fluorescent protein signals were detected with an Olympus fluorescence microscope (Japan). All primers used for gene cloning and vector construction were listed in Supplementary Table S1.

### 4.10. Plant Transformation and Phenotype Analysis

Arabidopsis was transformed with the *Agrobacterium* strain GV3103 mentioned above, according to the *A. tumefaciens* mediated floral dip method [57]. The transformants were identified based on kanamycin resistance, and the T_3_ generation seedlings were used for functional analyses. The flowering phenotype of different Arabidopsis lines, including *JrAHL2*-overexpressing (*JrAHL2-OE*), wild type (Col-0), and *AtAHL22* mutant (*ahl22-1*, Salk_018866), were identified according to the previous description [58]. To analyze the hypocotyl growth phenotype, Arabidopsis seeds were surface-sterilized and sown on Murashige & Skoog (MS) medium [56]. The plate was held at 4 °C for 3 days, exposed to white light for 4 h to stimulate germination, and then cultured for 6 days at 22 °C with a 16-h light/8-h dark photoperiod (light intensity of 150 μmol m^−2^ s^−1^) (long day) or darkness.

## 5. Conclusions

Thirty-seven *AHL* genes were identified in the walnut (*Juglans regia* L.) genome and found to be unequally distributed on 16 chromosomes. The phylogenetic tree divided *JrAHLs* into two clades. Segmental duplications were the major driving forces of *AHL* gene family expansion and evolution. Promoter and expression analysis indicated that *JrAHLs* were involved in multiple biological processes. Functional analysis showed that the nuclear-localized *JrAHL2* plays a negative role in floral transition and hypocotyl elongation. The first systematic exploration of the *AHL* gene family in walnut will provide helpful information for future work in walnut breeding.

## Figures and Tables

**Table 1:** Information of the *AHL* genes in *J. regia* L.

Name	Gene ID	Location (Start)	Location (End)	Strand	Length	CDS (bp)	Peptide (aa)	pI	MW (kDa)
*JrAHL1*	JreChr01G12984	39,611,176	39,611,994	+	819	819	272	6.08	28.02
*JrAHL2*	JreChr01G12992	47,390,686	47,391,612	−	927	927	308	6.59	32.84
*JrAHL3*	JreChr01G13333	47,470,900	47,477,455	+	6556	1005	334	9.59	34.79
*JrAHL4*	JreChr01G13761	50,056,338	50,057,327	−	990	990	329	5.08	34.44
*JrAHL5*	JreChr02G10898	7,160,919	7,167,725	−	6807	1035	344	9.91	35.36
*JrAHL6*	JreChr02G11467	21,601,290	21,602,144	+	855	855	284	6.45	29.38
*JrAHL7*	JreChr02G11474	26,318,938	26,319,843	−	906	906	301	6.80	31.86
*JrAHL8*	JreChr02G11687	26,371,944	26,376,879	+	4936	1068	355	8.83	36.83
*JrAHL9*	JreChr02G11978	27,859,904	27,860,884	−	981	981	326	5.60	34.15
*JrAHL10*	JreChr03G13350	4,426,812	4,430,966	−	4155	795	264	10.46	27.04
*JrAHL11*	JreChr04G10741	8,368,413	8,372,787	+	4375	1014	337	9.21	35.15
*JrAHL12*	JreChr04G12210	20,642,675	20,643,481	+	807	807	268	9.24	28.25
*JrAHL13*	JreChr06G10970	6,003,983	6,011,773	+	7791	930	309	9.61	32.44
*JrAHL14*	JreChr07G10387	23,875,413	23,876,234	−	822	822	273	9.74	28.01
*JrAHL15*	JreChr07G11230	16,643,389	16,644,297	−	909	909	302	5.77	31.30
*JrAHL16*	JreChr07G11239	27,147,611	27,148,277	−	667	666	221	10.09	23.04
*JrAHL17*	JreChr07G11616	27,252,429	27,256,966	+	4538	1107	368	10.09	36.49
*JrAHL18*	JreChr08G11927	30,383,998	30,384,978	+	981	981	326	7.07	34.60
*JrAHL19*	JreChr08G12220	5,871,308	5,872,264	−	957	957	318	6.36	33.92
*JrAHL20*	JreChr08G12225	8,400,985	8,408,164	−	7180	1182	393	10.33	39.80
*JrAHL21*	JreChr09G12239	8,457,851	8,459,672	+	1822	696	231	9.87	23.85
*JrAHL22*	JreChr10G11635	644,863	648,220	+	3358	1089	362	8.49	37.48
*JrAHL23*	JreChr11G10743	358,990	361,864	+	2875	1068	355	9.12	36.87
*JrAHL24*	JreChr11G11355	23,940,903	23,941,778	−	876	876	291	5.96	30.42
*JrAHL25*	JreChr11G11477	30,455,540	30,459,915	+	4376	1017	338	10.61	35.04
*JrAHL26*	JreChr11G11481	31,577,477	31,578,376	−	900	900	299	6.16	32.00
*JrAHL27*	JreChr12G10636	31,622,448	31,628,198	+	5751	939	312	9.63	31.89
*JrAHL28*	JreChr12G10638	18,010,455	18,017,606	+	7152	1134	377	9.37	38.78
*JrAHL29*	JreChr12G10640	18,021,521	18,027,959	−	6439	1140	379	9.59	38.92
*JrAHL30*	JreChr12G10998	18,039,195	18,040,085	−	891	891	296	6.10	31.07
*JrAHL31*	JreChr12G11092	21,387,711	21,392,430	+	4720	1020	339	10.00	35.22
*JrAHL32*	JreChr12G11095	22,118,819	22,119,700	−	882	882	293	6.30	31.42
*JrAHL33*	JreChr13G10464	22,173,675	22,179,704	+	6030	1221	406	9.59	42.63
*JrAHL34*	JreChr13G10846	1,739,954	1,740,790	−	837	837	278	5.80	28.67
*JrAHL35*	JreChr13G10963	23,001,456	23,006,865	−	5410	963	320	9.73	33.66
*JrAHL36*	JreChr14G10308	24,429,650	24,430,551	−	902	795	264	6.37	26.99
*JrAHL37*	JreChr01G12984	1,416,877	1,417,719	−	843	843	280	6.04	29.02

## Data Availability

All relevant data are available from the corresponding author on request (bdqgh@hebau.edu.cn).

## Supplementary Materials

The following supporting information can be downloaded at: https://www.mdpi.com/article/10.3390/ijms24087244/s1.
