# Supplementary material for: Systematical Characterization of the AT-Hook Gene Family in Juglans regia L. and the Functional Analysis of the JrAHL2 in Flower Induction and Hypocotyl Elongation
Source: Int J Mol Sci. 2023 Apr 14;24(8):7244. doi: 10.3390/ijms24087244 (PMC10138636; doi:10.3390/ijms24087244)
Supplement: Supplementary file 1 [file ijms-24-07244-s001.zip › Supplementary Table S1. JrAHL Primers were used in this study.pdf]

Supplementary Table S1. Primers were used in this study.

Gene name	primer name	primer sequence (5′ →3′)	purpose
JrAHL1	JrAHL1-qF	CTGTCGTAGCACTTCACGGA	RT-qPCR
	JrAHL1-qR	GGAACCTACCACGCTTCCTC	RT-qPCR
JrAHL2	JrAHL2-F	ATGGACCCAGTCACAGCAC	gene cloning
	JrAHL2-R	TTAATAAGGAGAGCGACCCGTG	gene cloning
	JrAHL2-2300F	ACGAGCTCGGTACCATGGACCCAGTCACAGCAC	vector construction
	JrAHL2-2300R	CATGGTGTCGACTCTAGAATAAGGAGAGCGACCCGTG	vector construction
	JrAHL2-qF	CGAAGTCGCTAATGGTTGCG	RT-qPCR
	JrAHL2-qR	GAAGCAGGTTGCCTTAGGGT	RT-qPCR
JrAHL7	JrAHL7-qF	GGTTGGTCCGCTTCTAGCAT	RT-qPCR
	JrAHL7-qR	CTGCTGTGACTGTTGTTGCC	RT-qPCR
JrAHL10	JrAHL10-qF	GTCATCCCAAAAGCGGGGTA	RT-qPCR
	JrAHL10-qR	AGTAAAACCCATCCCAGCCG	RT-qPCR
JrAHL11	JrAHL11-qF	CATGGCGACAGTTCAGCTTG	RT-qPCR
	JrAHL11-qR	CACTTCATTTGGCTGGCACC	RT-qPCR
JrAHL14	JrAHL14-qF	GAGCAGGTGGCTTGACGATA	RT-qPCR
	JrAHL14-qR	GAACTGGCGACTCTTCCTCC	RT-qPCR
JrAHL19	JrAHL19-qF	CCACAACAACAGCAACAGCA	RT-qPCR
	JrAHL19-qR	GGTTTGCGACTTTTGGGCTT	RT-qPCR
JrAHL28	JrAHL28-qF	AAATGGTGGCAGTCGTAGCA	RT-qPCR
	JrAHL28-qR	GCTGCCATTAGCATTCCTGC	RT-qPCR
JrAHL31	JrAHL31-qF	TTGGTGGTGCTGTTGCAATG	RT-qPCR
	JrAHL31-qR	GTGACTCTGAGCCTCCGAAC	RT-qPCR
JrAHL33	JrAHL33-qF	TTCGTATCAATTGCCGCACC	RT-qPCR
	JrAHL33-qR	TGGTATTGCGGATCTTCCTCG	RT-qPCR

Jr18SrRNA	Jr18S-qF	AGAACAGTCAGGGGCATTCG	RT-qPCR
	Jr18S-qR	ATCCCTCGTTGGCATCGTTT	RT-qPCR
AtFT	AtFT-qF	TGCAGGAATTCATCGTGTCGTGTT	RT-qPCR
	AtFT-qR	CTTCTTCCTCCGCAGCCACTCT	RT-qPCR
AtActin	AtACTIN-qF	GCGATTCCGTTGTCCTGAGGTTC	RT-qPCR
	AtACTIN-qR	TTCCACCACTGAGCACAATGTTACC	RT-qPCR
