# Supplementary material for: Systematical Characterization of the AT-Hook Gene Family in Juglans regia L. and the Functional Analysis of the JrAHL2 in Flower Induction and Hypocotyl Elongation
Source: Int J Mol Sci. 2023 Apr 14;24(8):7244. doi: 10.3390/ijms24087244 (PMC10138636; doi:10.3390/ijms24087244)
Supplement: Supplementary file 1 [file ijms-24-07244-s001.zip › Supplementary Table S2 Prediction of subcellular localization of JrAHL proteins.pdf]

Supplementary Table S2. Prediction of subcellular localization of JrAHL proteins.

Protein Name	Localization
JrAHL1	Nucleus
JrAHL2	Nucleus
JrAHL3	Nucleus
JrAHL4	Nucleus
JrAHL5	Nucleus
JrAHL6	Nucleus
JrAHL7	Nucleus
JrAHL8	Nucleus
JrAHL9	Nucleus
JrAHL10	Chloroplast
JrAHL11	Chloroplast
JrAHL12	Nucleus
JrAHL13	Nucleus
JrAHL14	Nucleus
JrAHL15	Nucleus
JrAHL16	Nucleus. Chloroplast.
JrAHL17	Nucleus
JrAHL18	Nucleus
JrAHL19	Nucleus
JrAHL20	Nucleus
JrAHL21	Nucleus
JrAHL22	Nucleus
JrAHL23	Nucleus
JrAHL24	Nucleus
JrAHL25	Nucleus. Chloroplast.
JrAHL26	Nucleus
JrAHL27	Nucleus
JrAHL28	Nucleus
JrAHL29	Nucleus
JrAHL30	Nucleus
JrAHL31	Nucleus. Chloroplast.
JrAHL32	Nucleus
JrAHL33	Chloroplast
JrAHL34	Nucleus
JrAHL35	Nucleus. Chloroplast. Mitochondrion.
JrAHL36	Nucleus. Chloroplast.
JrAHL37	Nucleus
