# Supplementary figures and images for: Systematical Characterization of the AT-Hook Gene Family in Juglans regia L. and the Functional Analysis of the JrAHL2 in Flower Induction and Hypocotyl Elongation
Source: Int J Mol Sci. 2023 Apr 14;24(8):7244. doi: 10.3390/ijms24087244 (PMC10138636; doi:10.3390/ijms24087244)

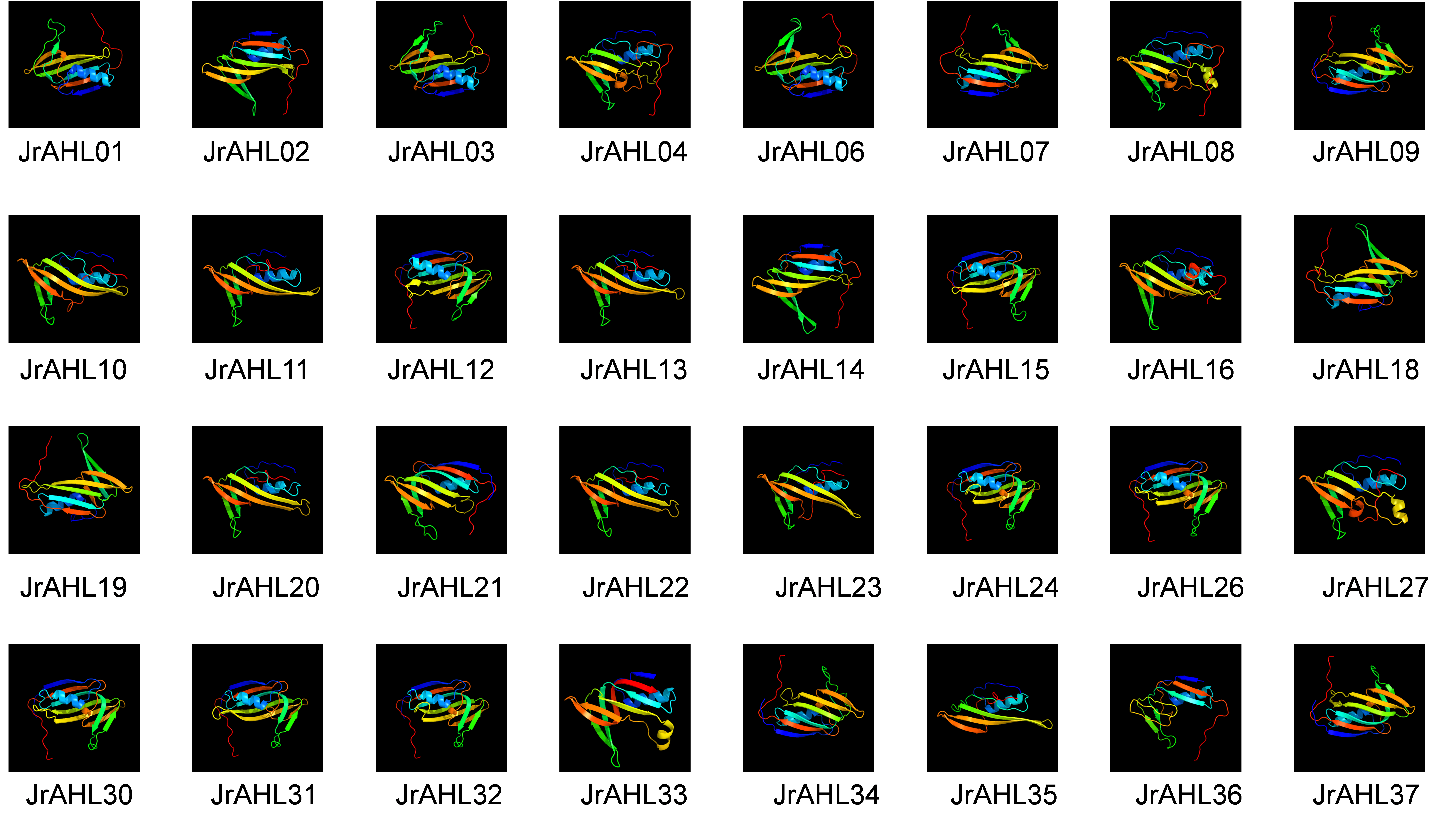

Supplement: Supplementary file 1 [file ijms-24-07244-s001.zip › Supplementary Figure S1. Predicted three dimensional structures of JrAHL proteins..tif]

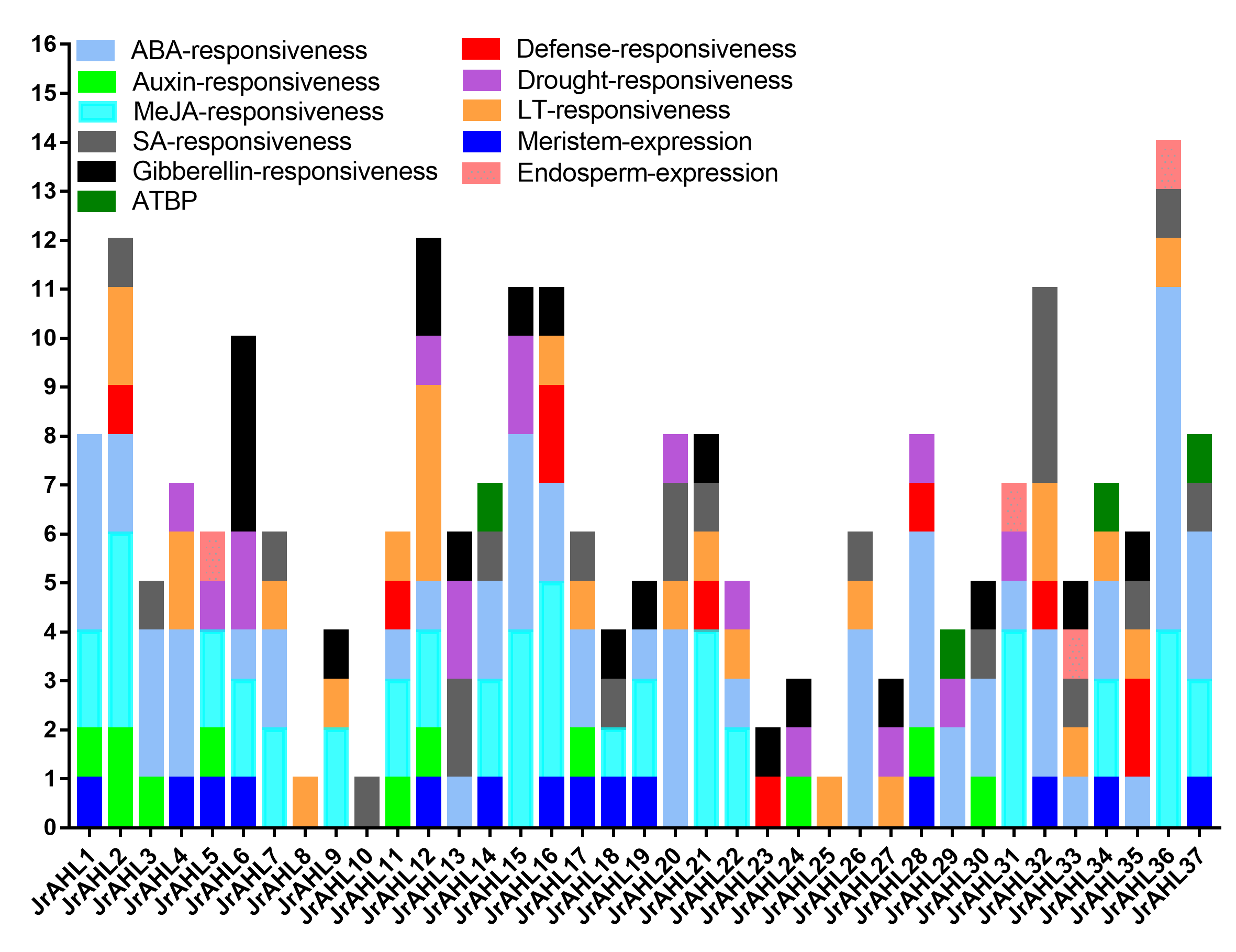

Supplement: Supplementary file 1 [file ijms-24-07244-s001.zip › Supplementary Figure S2. Predicted cis-elements in the JrAHL promoters.tif]
